# Evidence for placebo effects on physical but not on biochemical outcome parameters: a review of clinical trials

**DOI:** 10.1186/1741-7015-5-3

**Published:** 2007-03-19

**Authors:** Karin Meissner, Hans Distel, Ulla Mitzdorf

**Affiliations:** 1 Institute of Medical Psychology, Ludwig-Maximilians-University Munich, Munich, Germany

## Abstract

**Background:**

Recent reviews on placebo effects in clinical trials suggest that objective changes following placebo treatments may not exist or, at least, have been considerably overestimated. However, the possibility that yet unidentified subsets of parameters are responsive to placebo treatments has not been taken into account. Therefore, the aim of the present study is to examine the effects of placebo treatments on objectively measured outcome parameters by specifically focusing on peripheral disease processes.

**Methods:**

An initial dataset was collected from a MEDLINE search for placebo-controlled, randomized clinical trials. Trials with stable disease conditions were identified, and the effects of placebo treatments on peripheral outcome parameters were estimated by the changes from baseline in the placebo groups. An explorative data analysis was conducted in order to identify parameter classes with differential responsiveness to placebo treatments. A subgroup meta-analysis of a second dataset was performed to test whether the preliminary classification would also apply to placebo effects derived from the comparison of placebo groups with untreated control groups.

**Results:**

The explorative analysis of outcome parameters and strength of placebo effects yielded a classification into responsive "physical" versus non-responsive "biochemical" parameters. In total, 50% of trials measuring physical parameters showed significant placebo effects, compared with 6% of trials measuring biochemical parameters. A subgroup meta-analysis substantiated the differential response (physical parameters: *n *= 14, Hedges' pooled effect size g = 0.34, 95% CI 0.22 to 0.46; biochemical parameters: *n *= 15, g = 0.03, 95% CI -0.04 to 0.10). The subanalysis of the second dataset supported the classification and revealed a significant improvement for physical parameters (*n *= 20, g = 0.22, 95% CI 0.07 to 0.36) and a deterioration for biochemical parameters (*n *= 6, g = -0.17, 95% CI -0.31 to -0.02).

**Conclusion:**

The results suggest that placebo interventions can improve physical disease processes of peripheral organs more easily and effectively than biochemical processes. This differential response offers a good starting point for theoretical considerations on possible mediating mechanisms, and for future investigations in this field.

## Background

Since the introduction of double-blind randomized controlled trials, which have become the gold standard for assessing the efficacy of pharmacological treatments, reports on marked therapeutic changes in the placebo arms of the trials have led to the widespread belief that placebos have powerful effects [1]. However, in 1997, Kienle and Kiene [2] critically reviewed the placebo literature and concluded that the existing reports on powerful placebo effects do not withstand strong scientific criteria. Instead, most of the reported placebo effects could be explained by factors unrelated to the placebos, such as spontaneous improvement, additional treatment, or statistical regression to the mean. In accordance with other authors, they concluded that the "true" placebo effect [3] could best be identified by comparing the effects in the placebo arm of a study with those in an untreated control arm.

This approach was consequently followed by Hróbjartsson and Gøtzsche in their meta-analysis on clinical placebo effects. They analyzed 114 trials [4], and later an additional 44 trials [5,6], which contained both a placebo arm and a no-treatment control arm. Their analyses confirmed that the overall effect of placebo interventions, when compared with no intervention, is smaller than previously believed. They found a significant placebo effect only for subjective, patient-reported symptoms, most importantly pain. For observer-reported parameters, they did not find statistical evidence for a placebo effect. Slightly different predefinitions, however, do yield a small but significant placebo effect for observer-reported outcomes [7].

One plausible reason for the small or even absent placebo effect in these meta-analyses could be that placebo interventions do not affect all observer-reported outcomes equally. Instead, it might be that only a subset of outcome parameters is responsive to placebo treatment.

The aim of the present study was to examine whether placebo treatment can objectively improve peripheral disease processes. We focused on peripheral disease processes because evidence for placebo effects in this field is rather scarce. Therefore, we analyzed placebo effects on objectively measured outcome parameters from peripheral organs, tissues, and body fluids that were collected from two independent datasets of clinical trials.

The first dataset was derived from a MEDLINE search for placebo-controlled, clinical trials. Because the selected trials did not include untreated control groups, we restricted the dataset to trials on stable disease conditions, from which the effects of placebo treatments could be estimated by baseline changes within the placebo groups. We examined whether placebo treatments had differential effects on parameter subtypes and tried to establish an appropriate parameter classification. To test whether this preliminary classification would also apply to placebo effects derived from the comparison of placebo groups with untreated control groups, we added a subgroup meta-analysis of the trials collected by Hróbjartsson and Gøtzsche [6], using those with peripheral parameters as outcomes.

## Methods

### Definition of peripheral outcome parameters

Peripheral outcome parameters were defined as parameters measuring disease processes in peripheral organs, tissues, and body fluids.

### Analysis of placebo effects in clinical trials derived from a MEDLINE search

#### Trial identification and selection

The first dataset was derived from a literature search for placebo-controlled clinical trials published in five leading medical journals (*Annals of Internal Medicine*, *British Medical Journal*, *Journal of the American Medical Association*, *The Lancet *and *New England Journal of Medicine*) during the decade from 1991 to 2000. For this, a MEDLINE search was conducted by using the search-terms "placebo", "placebo(-)controlled" and "double(-)blind". This resulted in 1723 hits, among them 1689 double-blind, randomized trials using placebo controls. From these, all trials on non-clinical populations were excluded, as well as trials investigating a psychological, neurological or psychiatric disorder, or reporting exclusively on subjective symptoms. Trials lacking a classic placebo control group were also excluded. Furthermore, in order to select only trials with stable disease conditions, trials were excluded when the disease was expected to either improve or deteriorate during the study period, irrespective of experimental treatment, e.g., due to the natural course of the disease, or to co-interventions. For example, mild-to-moderate hypertension would be considered rather stable over a 4-week period in otherwise healthy patients but unstable in women developing hypertension during pregnancy. Similarly, hypercholesterolaemia would be considered rather stable over a 12-week period in patients maintaining their usual diet, but unstable when patients received dietary advice in addition to double-blind treatment. Thus, we searched for trials in which the baseline data provided an appropriate reference to interpret the changes observed within the placebo groups as the effect of the placebo intervention itself and not of other, placebo-unrelated factors. Finally, from the remaining trials, we excluded all trials lacking relevant details to determine the significance of baseline changes within the placebo groups. In total, 34 trials [8-41] investigating chronic stable clinical conditions and reporting on at least one peripheral outcome parameter fulfilled all criteria and were included in the analysis (Table 1).

#### Data extraction

From the 34 studies meeting the selection criteria, we extracted the baseline changes of peripheral outcome parameters within the placebo groups. When several parameters or time points had been measured, we analyzed the parameter or measurement with the largest effect size during the treatment period because we aimed at identifying outcome parameters most strongly affected by placebo treatment. From studies using a crossover design, we analyzed only the data of the group receiving placebo treatment first to avoid possible unblinding effects and drug carry-over effects.

#### Data analysis

For estimating the overall placebo effect on peripheral outcome parameters, we combined the effects of single trials by performing a meta-analysis based on 29 of the 34 trials. In five trials [10,21,31,36,37], data were not sufficient for the meta-analysis as only significance values of the effects of placebo treatment had been reported.

To start our explorative analysis, we determined the significance of the baseline changes within the placebo group of each trial. For this, we used the reported results of statistical testing or, when necessary, the means and measures of variability for performing two-tailed *t*-tests to estimate significance. We then identified possible common characteristics of parameters with significant or non-significant changes during placebo treatments. To substantiate the resulting dichotomous classification, we performed subgroup meta-analyses of the *n*-weighted standardized mean differences for each parameter subgroup.

In addition, to compare the efficacy of placebo relative to active treatment, we extracted the baseline changes of the analyzed parameters for the active treatment groups as well, and computed their *n*-weighted standardized mean differences. In cases with more than one active treatment arm, we selected the data of the most effective active treatment, according to study results. Placebo efficacy relative to active treatment was then estimated by dividing the standardized mean difference of the effect of placebo treatment by that of the active treatment.

### Analysis of placebo effects in clinical trials that included a no-treatment control group

#### Trial selection and data extraction

The second dataset for analyzing placebo effects on peripheral outcome parameters was collected from the updated review on placebo effects by Hróbjartsson and Gøtzsche [5,6]. First, we selected all 56 trials with observer-reported continuous outcomes. These included 13 trials with corresponding patient-reported and observer-reported continuous outcomes, from which only the patient-reported outcomes had been included in the main analysis by Hróbjartsson and Gøtzsche [6]. We excluded all trials on psychological, psychiatric or neurological conditions and those reporting exclusively on subjective symptoms, thereby selecting 26 trials that used peripherally measured parameters as outcomes [42-67]. Because the no-treatment groups controlled for unstable disease conditions due to natural or iatrogenic factors as well as for regression to the mean, this selection, in contrast to the first dataset, also included trials investigating acute or post-surgical conditions [46,47,59-62,67], as well as one trial in which subjects had not had a fully developed disease condition but were selected because of high normal blood pressure values [57], a procedure highly sensitive to the phenomenon of regression to the mean [68].

#### Data analysis

The results of the 26 trials were combined by calculating a meta-analysis of the *n*-weighted standardized mean differences for the post-intervention effects in the placebo and no-treatment groups. Subanalyses of parameter subgroups according to our classification were performed identically.

### Statistical analysis

For all meta-analyses, *n*-weighted standardized mean differences and 95% confidence intervals for the respective placebo effects were calculated using Hedges' adjusted effect size estimation and random effect models. To test for heterogeneity, χ^2 ^and I^2 ^statistics were performed. The computer program RevMan (version 4.2 for Windows) was used to calculate the meta-analysis measures. We allocated positive signs to effects favoring placebo treatment. For all statistical tests, *P *< 0.05 (two-tailed) was considered significant.

## Results

### Analysis of placebo effects in clinical trials derived from a MEDLINE search

All 34 placebo-controlled trials [8-41] meeting our inclusion criteria investigated pharmacological treatment, which was administered in 17 different clinical conditions (Additional file 1; Table 2, summarizing the data of the first dataset).

The meta-analysis of the baseline changes within the placebo groups of the 29 trials providing sufficient data showed a significant overall improvement of parameters during placebo treatment (Hedges' pooled effect size g = 0.21, 95% CI 0.12 to 0.31, *P *< 0.0001) with a high level of heterogeneity between studies (χ^2 ^= 63.37, *P *= 0.0001, I^2 ^= 55.8%).

The explorative analysis revealed that significant placebo effects were predominantly found for parameters measuring physical processes, e.g., blood pressure, or forced expiratory volume in 1 second (FEV_1_). Therefore, all types of physical outcome parameters were collected in one class, which was named "physical parameters". The remaining parameters, less frequently responding to placebo treatments, appeared to represent biochemical processes measured in peripheral body fluids and tissues, e.g., cholesterol and cortisol. Therefore, they were all taken together in one alternative class, which was named "biochemical parameters". To be more precise, 8 of 16 trials (50%) using physical parameters as outcomes reported significant placebo effects compared with only 1 of 18 trials (6%) using biochemical parameters. This difference was statistically significant (Fisher's exact probability test, *P *< 0.01).

**Figure 1:**
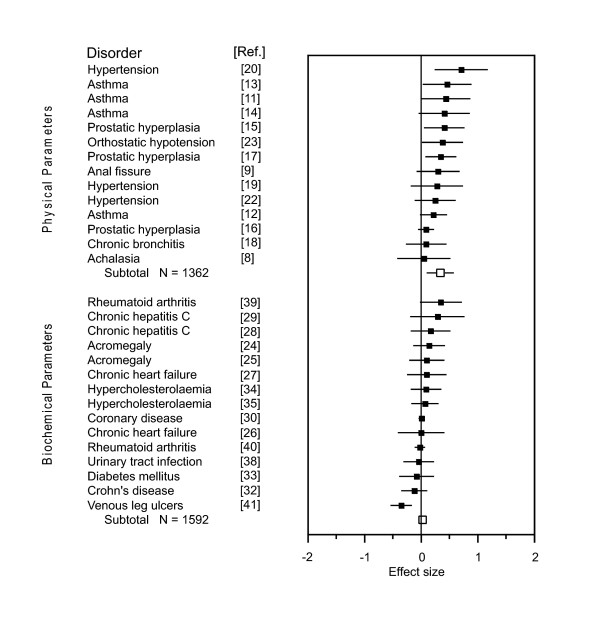
**Meta-analysis of the first dataset**. Standardized mean differences (placebo versus baseline) for physical and biochemical parameters in the 29 trials of the first dataset, retrieved from a MEDLINE search. Black squares indicate single effect sizes, white squares are the subgroups' mean effect sizes, and whiskers the 95% CI. Reference numbers are given in square brackets. Positive values favor placebo treatment.

To further substantiate this classification, we performed subanalyses for both groups of parameters. This revealed a significant placebo effect for physical parameters with a pooled effect size of g = 0.34 (95% CI 0.22 to 0.46, *P *< 0.0001), but an effect size close to zero for biochemical parameters (g = 0.03, 95% CI -0.04 to 0.10, *P *= 0.41) (see Figure 1). The differentiation between physical and biochemical parameters reduced heterogeneity (χ^2 ^= 24.63, *P *= 0.03, I^2 ^= 47.2%; and χ^2 ^= 6.39, *P *= 0.96, I^2 ^= 0%, respectively). Sensitivity analyses revealed that heterogeneity within the group of physical parameters was due to one outlier [20], and the exclusion of this study substantially reduced heterogeneity (I^2 ^= 0%).

All trials of the first dataset provided an active treatment arm. The median placebo efficacy ratio relative to active treatment in the total dataset was 0.26 (interquartile range (IQR) 0.05 to 0.37). For the subgroup of physical parameters it was 0.35 (IQR 0.27 to 0.42), or about one third of the active treatment effect, whereas for the subgroup of biochemical parameters, the ratio was close to zero (0.05; IQR -0.10 to 0.13). The difference between placebo efficacy ratios for physical and biochemical parameters was significant (Mann-Whitney *U *test, *P *< 0.001).

### Analysis of placebo effects in clinical trials that included a no-treatment control group

The dataset of Hróbjartsson and Gøtzsche [6] provided 26 trials with peripheral parameters as outcomes [42-67] (Additional file 2: Table 3, summarizing the data of the second dataset). In total, pharmacological treatment was investigated in 13 trials, physical treatment in 5, psychological treatment in 5, and placebo treatment in 3. These treatments were administered in 12 different disease conditions. The no-treatment control group was provided by a waiting-list control in one trial, and by all patients in seven trials using a crossover design.

**Figure 2:**
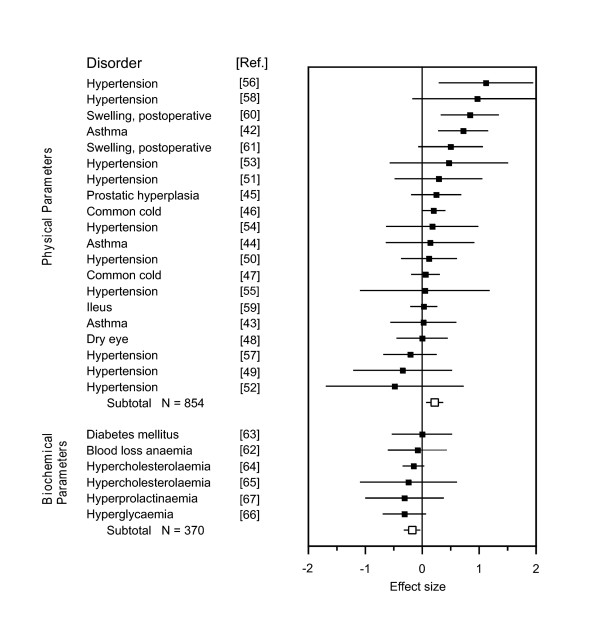
**Meta-analysis of the second dataset**. Standardized mean differences (placebo versus no treatment) for physical and biochemical parameters in the 26 trials of the second dataset, selected from a published review on placebo effects [6]. For details, see Figure 1.

The meta-analysis of all 26 trials failed to show a significant overall improvement due to placebo treatment (g = 0.11, 95% CI -0.01 to 0.24, *P *= 0.08), and heterogeneity between studies was high (χ^2 ^= 47.24, *P *= 0.005, I^2 ^= 47.1%). The application of our classification showed a significant improvement during placebo treatment for physical parameters (*n *= 20, g = 0.22, 95% CI 0.07 to 0.36, *P *= 0.003), whereas for biochemical parameters it showed the opposite (*n *= 6, g = -0.17, 95% CI -0.31 to -0.02, *P *= 0.02) (see Figure 2). There was slight heterogeneity within the group of physical parameters (χ^2 ^= 30.64, *P *= 0.04, I^2 ^= 38.0%), but not within the group of biochemical parameters (χ^2 ^= 1.36, *P *= 0.93, I^2 ^= 0%).

To confirm that the inclusion of acute and post-surgical conditions in our second dataset did not significantly influence the results, a reanalysis with these trials excluded was performed, thereby focusing on stable disease conditions. The effect sizes within the physical and biochemical parameter subgroups remained comparable with that of our main analysis (physical parameters: *n *= 15, g = 0.21, 95% CI 0.01 to 0.41, *P *= 0.04; biochemical parameters: *n *= 4, g = -0.17, 95% CI -0.32 to -0.01, *P *= 0.03).

## Discussion

Our results indicate that placebo interventions can improve objective measures of peripheral disease processes. They furthermore suggest that placebo interventions do not improve all kinds of peripheral outcome parameters equally, but primarily those reflecting physical disease processes.

The meta-analysis of placebo effects in the first dataset (collected from a MEDLINE search for placebo-controlled clinical trials) revealed a significant overall improvement of peripheral outcome parameters by placebo treatments. The explorative analysis indicated that significant effects occurred more frequently on physical than on biochemical parameters. Accordingly, an overall placebo effect across trials was only found within the subgroup of physical parameters. In comparison to the pharmacological medication, the administration of placebos improved physical parameters on average by one-third – a remarkable efficacy, not found for biochemical parameters. These results already suggest that placebo interventions affect physical parameters more frequently and strongly than biochemical parameters.

However, our classification was derived from clinical placebo-controlled trials without a no-treatment arm. These trials had not been designed to analyze placebo effects but to estimate the effect of the active medication against placebo control groups. Therefore, factors not due to placebo treatment may have contributed to the changes in the placebo groups in such trials, e.g., the natural course of the disease, and regression to the mean. We attempted to control for these factors by focusing on trials with otherwise stable disease conditions and tried to minimize the risk of regression to the mean by excluding trials on non-random samples selected by screening from a healthy population [68]. However, even in stable chronic conditions, symptoms may vary over time, and the possibility that some of the improvement on physical parameters may be due to regression cannot be fully excluded by the present data.

Therefore, to further substantiate our classification, we made use of the database of Hróbjartsson and Gøtzsche, which contains a complete collection of trials including both a placebo and a no-treatment control group [5,6]. Again, the subanalysis of trials with peripheral outcome parameters revealed a significant improvement from placebos compared with no treatment for the subgroup of physical parameters only. In fact, the analysis even showed a significant negative effect of placebos on biochemical parameters. However, as the number of trials reporting on biochemical parameters was small, this finding should be treated with caution.

A possible limitation in the present study is that physical and biochemical parameters were measured in different trials. Therefore, confounding variables, such as differences in study design or patient characteristics, may have biased the results. However, to use trials in which both parameters are measured in the same condition appears to be problematic, as the outcomes most probably would be coupled, i.e., any physical improvement would be expected to entail changes in biochemical parameters. Hence, our approach to compare trials with either type of parameter may provide the best available evidence to test the appropriateness of our classification. Furthermore, the fact that a remarkable difference in placebo effectiveness between physical and biochemical parameters was found in two different datasets renders the possibility of such a bias quite unlikely.

The responsiveness of physical parameters to placebo treatments becomes plausible when possible mechanisms mediating the effect are considered. Presumably, patients are able to monitor the state of their inner organs by sensory feedback – that is, by visceral perception [69] or by somatic perception (e.g. when experiencing respiratory effort in asthma [70] or ballistic movements in hypertension [71]). They may thus be able to monitor fluctuations in organ states rather quickly. During placebo treatment, the belief of the patient in being treated may result in selective attention to symptom improvement [72]. The momentary experience of symptom improvement may then act as a reward and positively reinforce preceding changes of autonomic function. Thus, visceral learning due to a mechanism similar to operant conditioning [73] may occur, in which the reward is internally provided [74]. The fast neural feedback from inner organs to cortical brain centers, which are involved in autonomic control [75,76], renders physical disease processes ideally suited for this kind of central modification.

Although many of the biochemical parameters are also modulated by the autonomic nervous system, there are several reasons for these being less placebo-sensitive. First, patients may have little or no visceral or somatic sensory feedback to monitor parameter changes, e.g., of insulin-like growth factor-1 or cholesterol, whether spontaneously occurring or induced by treatment. Second, even in cases when the actual concentration of a biochemical parameter can be estimated by the patient (e.g., blood glucose levels [77]) the nonspecific character of the symptoms on which these estimations may be based, and hence the difficulty in identifying spontaneous improvement, may prevent conditioning occurring. For example, varying blood glucose levels may affect thirst, hunger, and mouth dryness [77], symptoms that are also influenced by other factors, most importantly, food and water ingestion. Third, the latency between spontaneous parameter changes and the perception of improvement may be too long to provide contingent feedback [78], e.g., fibrinolytic activity in the healing of venous leg ulcers. Thus, most of the biochemical parameters may lack an essential element for placebo effects to be established, namely, the experience of momentary improvements due to autonomic changes.

A plausible explanation for the deterioration of biochemical parameters in the second dataset might be related to the diet-sensitivity of some of the parameters [63-66]: Patients in placebo groups, believing they are being treated, may become less cautious in following their diet, while untreated patients, necessarily knowing about the lack of treatment, may pay more attention to their diet. Alternatively, an unblinding of placebo recipients during the trial may lead to frustration and therefore to less compliance with the dietary regimen.

One frequently discussed mediating mechanism of placebo effects is the patient's expectation of clinical improvement, which can be raised, for example, by verbal suggestions accompanying placebo treatment [79,80]. Expectation and operant conditioning may complement each other. Learning theory emphasizes the importance of response-specific expectations for the performance of operant conditioning tasks [81]. Thus, the patient may direct his/her attention to symptom improvement because he/she is expecting a clinical benefit. In this sense, expectation may be necessary to both initiate and maintain the process of operant conditioning. It has recently been demonstrated that in patients with parkinsonian disease, both the expectation and the actual experience of a clinical benefit during placebo treatment activates the inner-brain reward circuitry [74,82]. This experimental result fits well with the hypothesis that both mechanisms (expectation and operant conditioning) are involved in the mediation of placebo effects.

Classical drug conditioning is also considered one of the mechanisms mediating placebo effects [79,80]. Classically-conditioned drug effects are, however, expected to extinguish after repeated administration of an inactive agent, and therefore should diminish over the course of clinical trials, in which treatment usually is administered repeatedly [83]. Positive outcomes in the placebo groups of clinical trials are therefore more likely to be induced and maintained by expectation rather than by classical conditioning.

Notably, there is evidence from experimental studies that expectations raised by placebo suggestions can affect physical parameters of peripheral organs, e.g., gastric and pulmonary function [84-86], but not biochemical parameters, i.e., growth hormone and cortisol [87]. These experimental results lend support to the present result that physical and biochemical parameters respond differentially to placebo treatments, with only physical parameters being affected by expectation, and thus displaying placebo effects in clinical trials.

In this study, we were able to identify different kinds of peripheral outcome parameters as one reason for the heterogeneity of placebo effects on observer-reported outcomes. The differential placebo responsiveness of physical versus biochemical parameters should be taken into account when designing future pharmacological studies. A question that ties in with the present results concerns the putative specificity of placebo effects. Depending on the type of disease (e.g., psychiatric, neurological, internal), on the information delivered to the patient, and on the patient's former experiences with treatments, the effects of placebo interventions may differ, and different mechanisms may be involved. To disentangle the specific components of placebo treatments under different circumstances and to work out their effects represents a major challenge for future placebo research.

## Conclusion

Our results indicate that placebo treatments of peripheral disease processes can affect physical parameters more easily and strongly than biochemical parameters. This differentiation holds true for both datasets we tested, i.e., conventional placebo-controlled clinical trials, and clinical trials that included a no-treatment arm. As a corollary, it follows that placebo-responsive subgroups may also be identified in datasets in which global averages conceal such specific responses.

Although much progress has been made in the past decade in understanding the biological basis of placebo effects in neurological conditions, e.g., pain and parkinsonian disease, the mechanisms that mediate placebo effects on peripheral organ systems still await to be further elucidated. The differential placebo responsiveness of physical versus biochemical parameters, as disclosed in the present study, offers a good starting point for theoretical considerations on possible mediating mechanisms, as well as for future investigations in this field.

## Competing interests

The authors declare that they have no competing interests.

## Authors' contributions

KM designed the study, collected, prepared and analyzed the data, performed the statistical analysis, interpreted the data and drafted the manuscript. HD and UM participated in the design of the study and in the interpretation of data and helped to draft the manuscript. All authors read and approved the final manuscript.

## Pre-publication history

The pre-publication history for this paper can be accessed here:



## Supplementary Material

Additional File 1 Parameters of trials contributing to the physical and biochemical subgroups of the first dataset (placebo versus baseline) Click here for file

Additional File 2 Parameters of trials contributing to the physical and biochemical subgroups of the second dataset (placebo versus no treatment) Click here for file

## Figures and Tables

**Table 1:** Reasons for exclusion and number of excluded trials during the selection process of the first dataset

**Reasons for exclusion**	***n***
**Lack of placebo-controlled, double-blind, randomized trial**	
Review, article, or comment	272
No placebo used	186
Lack of double-blinding or randomization	96
**Kind of investigated symptom or condition**	
No clinical condition	44
Psychological, neurological or psychiatric condition	106
Subjective symptom	67
**No classic placebo control group**	
Placebo as a dummy to maintain blinding of experimental treatments	147
Placebo as a control of an add-on treatment	74
**Lack of stable disease condition**	
Medical intervention preceding experimental treatment	120
Co-medication changed during study	33
Acute condition	79
Developmental disorder	20
Trial focus on relapse or prevention	131
Trial focus on death or other serious events	113
Trial focus on long-term development of disease	121
**Miscellaneous**	
Crossover design and results not separated by sequence	23
Native physiological parameter not reported	17
Insufficient detail to estimate placebo effect	40
**Total**	1689
